# Regulation of the brain tumor microenvironment by focused ultrasound

**DOI:** 10.1016/j.omton.2025.200994

**Published:** 2025-05-14

**Authors:** Kang Fu, Huijing Hu, Xiaodong Zhou, Le Li, Li Yan

**Affiliations:** 1 Institute of Medical Research, Northwestern Polytechnical University, Xi’an 710072, China; 2 Ultrasound Diagnosis & Treatment Center, Xi’an International Medical Center Hospital, Xi’an 710100, China

**Keywords:** MT: Regular Issue, ultrasound, brain tumors, microenvironment, focused ultrasound, tumor microenvironment, TME, regulation, diagnosis, therapy

## Abstract

Glioblastoma and other high-grade primary malignant brain tumors are a serious threat to the life and health of patients; consequently, their accurate diagnosis and treatment are crucial. Brain tumors are usually treated by surgical resection, radiotherapy and drug chemotherapy; however, such treatments have side effects such as trauma, infection, and radiation exposure. Furthermore, owing to limitations in conditions such as the skull and blood-brain barrier, noninvasive treatment and diagnosis of brain tumors have been challenging. In recent years, focused ultrasound (FUS) technology has shown great advantages and application potential because of its noninvasive and energy-focusing characteristics in brain tumors. From the perspective of the brain tumor microenvironment, FUS can produce mechanical and thermal effects by delivering sound waves to brain tissue; these sound waves can induce blood-brain barrier opening, radiation sensitization, targeted substance delivery, immune enhancement, angiogenesis and destruction, oxidative stress, interstitial hydraulic regulation, and brain tumor marker sonobiopsy. The feasibility and safety data from both animal models and clinical trials support FUS as having great potential for use in the diagnosis and treatment of brain tumors.

## Graphical abstract

**Figure:**
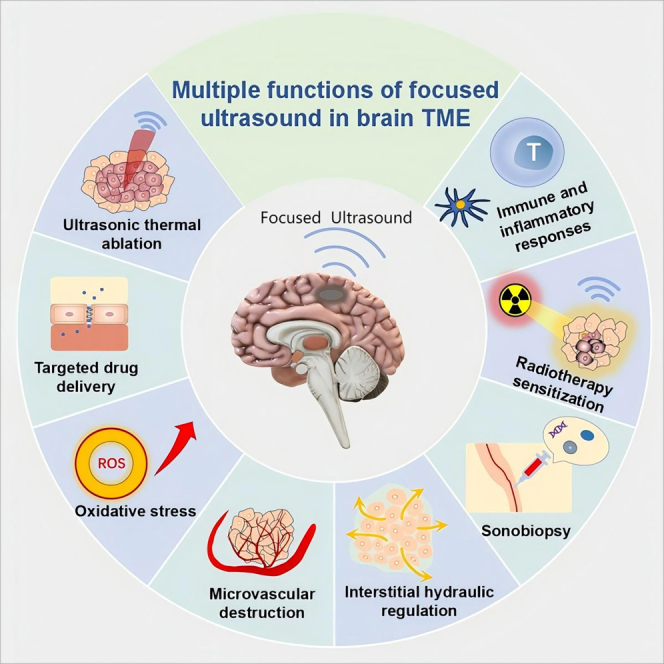

## Teaser

Yan and colleagues explore the innovative use of focused ultrasound (FUS) technology for the regulation of the brain tumor microenvironment. They highlight FUS’s ability to noninvasively enhance therapeutic outcomes, demonstrating its significant potential to address challenges posed by the blood-brain barrier and improve treatment efficacy in brain tumors.

## Introduction

Glioblastoma and other high-grade primary malignant brain tumors are a serious threat to the life and health of patients, and their accurate diagnosis and treatment are crucial. The current standard therapy typically involves surgery, radiotherapy, and chemotherapy.^1^^,^^2^ Invasive surgical therapy is not suitable for all brain tumor patients because of the large amount of trauma, high surgical recurrence rate, and complexity of the nervous system,^3^^,^^4^^,^^5^ and it is also associated with the risk of bleeding and infection. Owing to the location of the tumor, the blood-brain barrier (BBB), and the blood-tumor barrier (BTB), the delivery of chemotherapeutic drugs into brain tumors is limited,^6^ affecting their efficacy. Moreover, radiotherapy is neurotoxic, and high doses of radiation can damage normal brain tissue, limiting the exposure dose.^7^ Consequently, there is an urgent need for new noninvasive, safe, and widely applicable clinical treatment methods.

**Figure 1:**
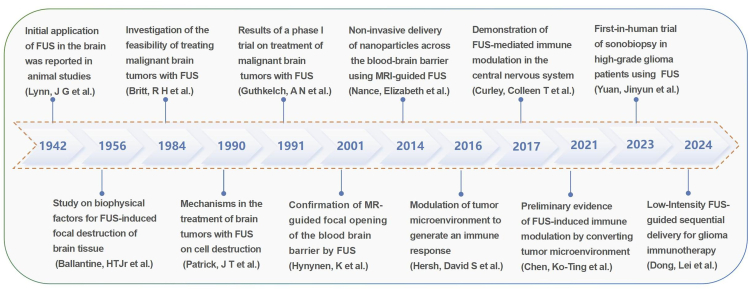
The timeline of the historical progress of focused ultrasound in the application of the brain tumors, covering significant milestones and representative papers from early applications to recent research (FUS, focused ultrasound)

Ultrasound is a noninvasive treatment method that is used in clinical settings to treat various conditions. Unlike traditional ultrasound, focused ultrasound (FUS) typically involves the use of a concave transducer, a lens, or a phased array to focus the ultrasound waves to a focal tissue,^8^ resulting in a high energy density.^9^ FUS exhibits precision and noninvasiveness, producing both thermal and non-thermal effects during treatment. The thermal effect is realized through the targeted heating of tissues, leading to cell death and tumor ablation, which renders it particularly effective for treating refractory brain tumors. This application has been the focus of extensive preliminary research. The non-thermal effects encompass mechanical vibrations induced by ultrasound, enhanced cell membrane permeability, and the activation of signaling pathways. These non-thermal effects hold significant potential for drug delivery and the modulation of the tumor microenvironment (TME). In recent years, with the increasing prevalence of minimally invasive methods, the exploration and application of FUS for the treatment of brain tumors have received increasing attention,^10^^,^^11^^,^^12^^,^^13^^,^^14^^,^^15^^,^^16^^,^^17^^,^^18^^,^^19^^,^^20^^,^^21^ as showed in the timeline (Figure 1).

At present, numerous studies have reported that inflammation in the brain TME, BBB disruption, oxidative stress, tumor vascularization, and interstitial hydraulic pressure are involved in the mechanisms by which FUS affects cancer. Moreover, the identification of brain tumor markers in the microenvironment from liquid biopsy samples and drug delivery experiments have aided in the elucidation of these mechanisms. The purpose of this review is to summarize the therapeutic effects of transcranial FUS on the microenvironment of brain tumors and to provide a comprehensive reference and promote its translation to clinical practice.

## Epidemiology

Central nervous system (CNS) tumors, including brain tumors and spinal cord tumors, are the second most common cancers in teenagers and young adults.^22^ Brain tumors can be divided into benign brain tumors and malignant brain tumors. Among them, malignant brain tumors have a high degree of malignancy, are difficult to be completely cured, and have a poor prognosis. According to statistics, glioblastoma is the most common type of malignant brain tumor, accounting for approximately 49% of brain cancer cases, followed by diffuse low-grade glioma (30%). The 5-year survival rate for glioblastoma patients is even lower than 10%.^23^ In addition, brain tumors exhibit considerable cellular and molecular heterogeneity not only between patients but also within their TME itself^24^^,^^25^^,^^26^^,^^27^^,^^28^; for example, glioblastoma can be divided into dozens of subgroups,^29^^,^^30^ which poses greater requirements for the diagnosis of brain tumors. Early intervention can improve patient prognosis.^31^ This review specifically focuses on World Health Organization grade III/IV gliomas (including astrocytoma isocitrate dehydrogenase (IDH)-mutant and glioblastoma IDH-wild type), diffuse midline glioma H3 K27-altered, and medulloblastoma as representative malignant brain tumors. The heterogeneity of these entities necessitates precise targeting strategies. Therefore, early and accurate diagnosis and treatment of brain tumors are very important. However, the diagnosis and treatment of brain tumors are limited by factors such as the skull, BBB, BTB, and other factors. There is an urgent clinical need for noninvasive techniques for the diagnosis and treatment of brain tumors.

## Brain TME

The cellular microenvironment mainly refers to the physical and chemical environment around brain tumors and includes the biological microenvironment (immune cells, blood vessels, extracellular matrix [ECM], etc.), chemical microenvironment (reactive oxygen species [ROS], pH, temperature, etc.), and physical microenvironment (ECM biomechanics, interstitial fluid pressure, etc.). As tissues exhibit abnormal proliferation, tumors have a unique cellular microenvironment—the TME. It is composed mainly of tumor cells and their surrounding immune and inflammatory cells, lymphocytes, tumor-associated fibroblasts, ECM, microvessels, and various cytokines and chemokines^32^ and is a complex comprehensive system. The brain TME has the following characteristics: (1) hypoxia,^33^^,^^34^ (2) acidic pH,^35^ (3) angiogenesis,^36^ (4) hardening of the ECM, (5) high interstitial hydraulic pressure,^37^ (6) immunosuppression, (7) abnormal redox levels,^38^ (8) chemotherapy resistance,^39^ and (9) BBB and BTB.^40^ The TME provides protection and support for the survival and dynamic development of brain tumors affording them the characteristics of drug resistance, heterogeneity and immunosuppression.^39^^,^^41^^,^^42^^,^^43^ Thus, the regulation and destruction of the immunosuppressive TME are critical for the diagnosis and treatment of brain tumors.^44^^,^^45^^,^^46^^,^^47^^,^^48^^,^^49^^,^^50^

## Blood-brain barrier

The BBB is a unique characteristic of the brain TME. The BBB is composed mainly of brain capillary endothelial cells connected by tight junctions,^51^ which maintain the stability of the neural microenvironment by regulating molecule and ion transport between the brain and blood.^52^ In the TME, owing to the increased demand for oxygen and nutrients, there is a special barrier between the newly synthesized microvasculature and the surrounding tumor, namely, the BTB.

When combined with microbubbles, low-intensity focused ultrasound (LIFU) can increase the permeability of the BBB and BTB,^17^^,^^53^^,^^54^ and changes in tight junctions have been observed via electron microscopy.^55^ Moreover, the intracellular levels of occludin, claudin-5, and ZO1 decrease.^55^^,^^56^ The specific mechanism may be related to the increase in the shear stress of endothelial cells caused by FUS. Changes in shear stress can regulate the endothelial phenotype, and the stabilization of endothelial barrier function in brain microvessels involves vascular endothelial (VE)-cadherin signaling.^57^ A mechanosensory complex consisting of vascular endothelial growth factor (VEGF)-R2, VE-cadherin, and platelet endothelial cell adhesion molecule (PECAM)-1 can be activated by PI3K *in vitro* to regulate various pathways. Relevant animal studies have shown that FUS increases both the phosphorylation of protein kinase B (Akt) and the permeability of the BBB through the PI3K/Akt pathway.^58^

The opening of the BBB and the BTB allows the exchange of larger molecules between the brain TME and the blood. Delivery systems are currently used to increase the targeted delivery and concentration of substances such as brain tumor drugs, and these systems are also involved in the identification of information molecules in the brain TME.

## Tissue ablation

As a noninvasive diagnostic and treatment procedure that uses sound wave energy to induce changes in the brain, FUS can be divided according to the energy intensity into LIFU and high-intensity focused ultrasound (HIFU). HIFU is generally used for tumor ablation, whereas LIFU is often used for microenvironmental regulation. The key distinctions between the two modalities regarding their brain applications in the treatment of brain tumors are summarized in Table 1.

**Table 1:** Comparative analysis of HIFU and LIFU in brain applications

Feature	HIFU (high-intensity focused ultrasound)	LIFU (low-intensity focused ultrasound)
Primary applications	mainly used to directly ablate diseased tissue	used for non-invasive brain stimulation, neuromodulation, and drug delivery
Probe design	larger probes designed for focusing and generating high energy output	smaller probes designed for low energy output and fine control
Intensity	high, 500–10,000 W/cm^2^	low, 0.1–1.0 W/cm^2^
Temperature	55°C–65°C	<37°C
Tissue response	causes significant thermal damage and tissue necrosis	primarily elicits mild responses through mechanical wave effects
Treatment duration	typically short, capable of rapidly producing therapeutic effects	requires longer exposure times and more frequent occurrences to achieve desired biological effects
Safety	requires careful energy control to avoid damage to surrounding normal tissue	generally considered to have high safety, with fewer side effects
Imaging and guidance	requires real-time imaging techniques (e.g., MRI and ultrasound) for treatment guidance	may utilize imaging techniques but with lower precision requirements

Tissue ablation by FUS can be divided into thermal ablation and mechanical ablation. When an ultrasound wave passes through biological tissue, it is converted to heat energy, increasing the tissue temperature.^59^ This process can cause coagulation necrosis.^60^ Compared with traditional craniotomy, thermal brain tumor ablation can be used to avoid the risks of bleeding and infection.^61^ Studies have shown that, at temperatures higher than 55°C, hyperthermia can lead to coagulation necrosis, protein denaturation, and destruction of tumor cell membranes.^62^^,^^63^ FUS-ablation guidance usually includes ultrasonic guidance and magnetic resonance guidance, and magnetic resonance guidance is often used in current studies. Magnetic resonance imaging (MRI)-guided FUS (MRgFUS) is a noninvasive thermal ablation technique. The mechanical energy of ultrasound is absorbed by the tissue at the focal point and converted to heat energy, resulting in local destruction of the tissue.^64^ Accurate target positioning and heat detection are achieved via MRI.^65^ In 2014, Coluccia et al. used MRgFUS for the first time to perform noninvasive thermal ablation of a centrally located recurrent glioblastomas in patients.^59^ No adverse events or neurological deficits were reported. Mechanical ablation is related to the cavitation effect, mainly inertial cavitation, which can mechanically disintegrate tissues and is often used for tissue fragmentation. The target tissue is transformed into liquid debris by a high-intensity short ultrasound pulse that can be absorbed by the body.^66^ Tissue fragmentation has been shown to effectively destroy the brain TME and ablate glioma in mice.^67^

## Radio sensitization

FUS can increase the sensitivity of brain tumor cells to radiation therapy, resulting in a reduced required radiation dose and associated side effects and improved efficacy. Previous studies have shown that hyperthermia sensitizes glioma stem cells to radiation therapy by downregulating members of the Akt signaling pathway.^68^ Interstitial microwave hyperthermia combined with radiation therapy can prolong the overall survival of patients with high-grade gliomas.^69^

The combination of microbubbles and ultrasound can enhance the therapeutic effect of ultrasound therapy and lower the required radiation dose. Recent animal studies have shown that low-dose radiation therapy combined with microbubble ultrasound can improve the efficacy of radiotherapy for glioblastoma by increasing DNA damage.^70^ In addition, He et al. proposed that ultrasound microbubbles could increase the radiosensitivity of glioblastoma by inhibiting tumor autophagy mediated by progesterone receptor membrane component 1.^71^ Hypoxia enhances radioresistance by reducing ROS levels. Numerous studies have shown that increased blood-brain cell membrane pore permeability caused by FUS can promote oxygen delivery and perfusion in the TME^72^^,^^73^ and may also serve as a mechanism of radiosensitization in brain tumors.

Chen et al. recently demonstrated the synergistic effect of FUS with radiotherapy in a mouse model of GL261.^74^ In another animal study, histological analysis of gliomas 72 h after FUS combined with radiotherapy (4 Gy) revealed 93% and 396% increases in apoptosis, respectively, compared with that after FUS and radiotherapy (RT) alone.^75^ However, other doses have resulted in limited improvement, so the optimal dose still needs further research. Tazhibi et al. demonstrated the feasibility and safety of FUS-mediated BBB opening in the treatment of diffuse midline glioma of the brain stem with moderately hypofractionated radiotherapy.^76^ In addition, clinical evidence from an interim analysis of an ongoing clinical trial (NCT01628406) indicates that the combination of radiotherapy and FUS yields no FUS-related adverse effects in recurrent malignant high-grade glioma patients. This characteristic may facilitate clinical applications.

## Drug delivery

**Figure 2:**
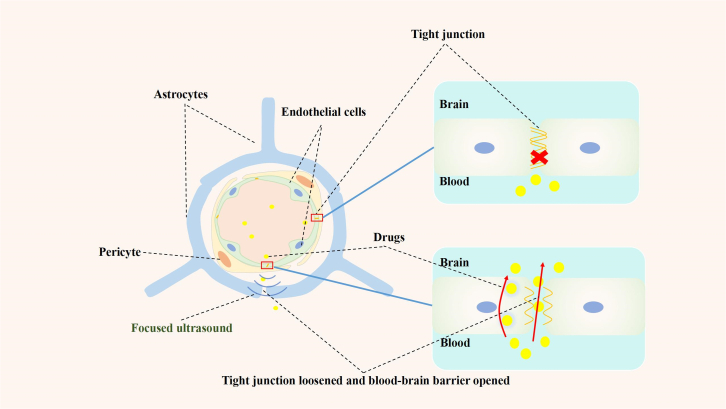
Schematic diagram of drug delivery mediated by focused ultrasound Under irradiation via focused ultrasound, the tight junctions of endothelial cells in the blood-brain barrier became loose and opened reversibly for a short time, which promoted the entry of drugs into the brain tumors and increased their concentrations within these neoplasms.

The BBB and BTB severely impede drug delivery to the CNS,^77^ which is an obstacle for effective brain tumor treatment. The reversible opening of the BBB and the opening of the BTB mediated by FUS enhance the delivery of various drugs (Figure 2), such as etoposide,^78^ panobinostat,^79^ temozolomide,^80^ paclitaxel,^81^ and carboplatin,^82^^,^^83^ to brain tumors, significantly increasing the concentration of the drug in the brain TME, thereby inhibiting tumor growth and prolonging survival.

With the in-depth study of FUS-mediated BBB opening, ultrasound delivery is not limited to chemotherapeutic drugs, and FUS can also enhance the transport of other anticancer molecules, such as genes,^84^^,^^85^^,^^86^ viruses,^87^ cells,^88^^,^^89^ antibodies,^90^^,^^91^ nanoparticles,^92^ recombinant adenoviral vectors,^93^ and immune checkpoint inhibitors,^94^ to improve the brain TME. For example, in the presence of FUS, B-cell-derived exosomes (B-Exo) macrophage-derived exosomes can effectively aggregate in glioma cells and effectively inhibit the growth of gliomas without significant adverse reactions.^95^ In addition, after FUS radiation, Exos secreted by mesenchymal stem cells as vectors of the tumor suppressor gene miR-1208 promote the uptake of miR-1208 by gliomas, thereby inhibiting the expression of NUP214 and the activity of the transforming growth factor (TGF)-β pathway and achieving highly efficient cancer inhibition.^95^ In a medulloblastoma model, the targeted delivery of small interfering RNA to the microenvironment by microbubbles (MB)-FUS reduces smoothened (SMO) protein production and significantly increases tumor cell death.^96^ Yang et al. used MB-FUS for the targeted delivery of CRISPR-Cas9 plasmids encapsulated in lipid nanoparticles, which increased the sensitivity of mouse glioblastoma cells to temozolomide and prolonged survival.^97^ A clinical study of 4 patients with brain metastases from breast cancer (NCT03714243) revealed for the first time that MRgFUS could noninvasively deliver monoclonal antibodies across the BBB^98^ to the human brain.

## Immunity and inflammation

**Figure 3:**
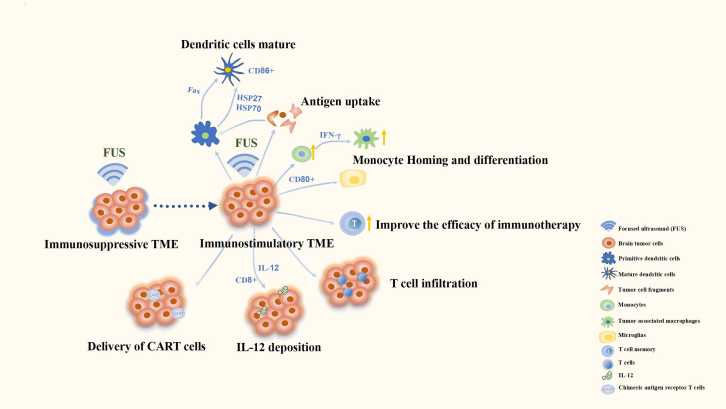
Focused ultrasound and the immune microenvironment of brain tumors Focused ultrasound stimulation contributes to the formation of an immunostimulatory tumor microenvironment. Moreover, focused ultrasound can promote the transformation from an immunosuppressive microenvironment to an immunostimulatory microenvironment under specific circumstances.

Brain tumors are defined as cold tumors.^99^ The brain TME shows reduced T cell infiltration and low natural killer cell infiltration.^100^ Tumor-associated neutrophils are associated mainly with the tumor-promoting N2 phenotype^101^ and other immunosuppressive features. Because brain tumors are protected by the BBB, BTB, and vascular heterogeneity, immunotherapy approaches mainly include targeted delivery of immune substances and activation of the immune microenvironment (Figure 3). In recent years, the application of FUS in the immunotherapy of brain tumors has been verified by numerous experiments.

In normal brain tissue, BBB opening mediated by microbubbles can cause the acute release of proinflammatory factors and chemokines from cells, inducing rapid sterile inflammation,^102^ which may alter the immune microenvironment. First, the mechanical destruction of tumors releases a large amount of tumor-associated debris and antigens, which activate dendritic cells.^103^ In terms of promoting antigen presentation, HIFU increases the concentration of interferon-γ while decreasing the concentration of interleukin (IL)-10 and maintaining the concentrations of IL-4, TGF-β1, and TGF-β2 in mouse neuroblastoma cells.^104^ High-grade glioma animal experiments have shown that FUS can enhance antibody uptake in brain tumor-targeted regions.^90^ In addition, in tumors that have been treated with MRgFUS, the number of monocytes and monocyte-derived tumor-associated macrophages increases, and the number of CD80^+^ cells among monocytes and microglia increases, suggesting that MRgFUS activates monocytes in the glioma cell microenvironment. Homing and differentiation of the immune microenvironment leads to a more proinflammatory state.^105^

FUS can promote the delivery of immunotherapeutic agents and drugs to improve the immune microenvironment. Some studies have shown that MB-mediated FUS intervention can enhance the function of proinflammatory macrophages, promote the formation of long-lived memory T cells in the brain, and improve the efficacy of immune checkpoint blockade in glioblastomas.^106^ IL-12 can exert antiglioma effects by stimulating the tumor immune response. MB-FUS-induced BBB opening can increase the deposition of IL-12 in the brain and increase the CD8^+^/regulatory T cells (Treg) ratio, thereby inhibiting brain tumor growth.^107^ LIFU enhances the delivery of chimeric antigen receptor T-cell (CAR-T) cells to the glioma microenvironment, promotes the infiltration of T cells into the glioma TME, and significantly improves the survival rate of experimental mice.^108^ However, the immune response caused by FUS may be dose dependent. Some studies have shown that the immune response in humans has not been observed at the clinical FUS intensity. In animal experiments, a higher dose within the safe range could recruit lymphocytes into the TME, thereby switching the immunosuppressive TME to an immunostimulatory TME in glioma.^12^

## Vasculature

Owing to the spatial and temporal heterogeneity of blood vessel permeability in the TME,^109^ the delivery of drugs to the^110^ microenvironment is affected. Therefore, improving vascular permeability in the TME is helpful for the drug treatment of tumors. Studies have shown that LIFU combined with microbubbles can induce stable cavitation, cause mechanical detachment of endothelial cells, and enhance microvascular permeability.^111^ Moreover, HIFU combined with microbubbles has been found to enhance the antivascular effect and penetration efficiency of drugs in the tumor core and at its edge.^112^ FUS-mediated BBB/BTB opening significantly increased stromal tumor blood flow and enhanced the delivery of nanoparticles to the brain TME.^85^

HIFU assisted by microbubbles can also cause damage to blood vessels via inertial cavitation. Excessive dilation or invagination of the microvasculature can be caused by high shear stress and temperature in local areas.^113^ Studies have shown that acute and complete interruption of blood flow can occur within seconds after ultrasound treatment.^114^ The appropriate destruction of microbubbles by ultrasound to selectively deplete the tumor neovasculature can significantly reduce tumor blood perfusion and microvessel density, promote the necrosis and apoptosis of tumor cells, delay tumor growth, and improve the survival rate of experimental mice.^115^ In a subcutaneous animal model of glioma, low-duty cycle ultrasound combined with microbubbles significantly reduced blood flow in the microenvironment and inhibited tumor growth.^116^

## Interstitial hydraulic pressure

The mechanical force generated by the ECM plays a key role in cancer progression.^117^ Malignant gliomas form a neovasculature through the stimulation of VEGF. These blood vessels are structurally and functionally abnormal, resulting in high interstitial hydraulic pressure in the microenvironment.^37^ Ultrasound may reduce high interstitial fluid pressure in brain tumors via the following mechanisms. First, the thermal effect of ultrasound causes an increase in local blood flow.^118^ Vascular permeability may be enhanced after FUS intervention^119^ because of the tissue displacement caused by the acoustic radiation force and momentum transfer.^120^^,^^121^ Pore openings can be observed at the cellular level, causing the fluid to flow toward the tumor edge and reducing the interstitial fluid pressure in the microenvironment.^122^ In addition, the cavitation of FUS can lead to the separation of collagen fibers and destruction of the dense collagen matrix.^123^ The denaturation temperature of collagen in the microenvironment decreases under acidic and hypoxic conditions.^124^^,^^125^^,^^126^ The thermal effect of ultrasound can cause denaturation and unfolding of the collagen structure, resulting in an increase in the spacing between fibers.^127^ This process enhances the outwards flow of fluid in the TME and reduces the high interstitial fluid pressure (IFP).^128^

## Oxidative stress

Hypoxia, high glutathione levels, and high ROS levels are characteristic of the brain TME. Ultrasound-based sonodynamic therapy (SDT) has recently been shown to increase oxidative stress in tumor cells. SDT triggers sound sensitization mainly through ultrasound, resulting in an increase in ROS levels and cavitation, inducing tumor cell apoptosis or even directly killing tumor cells.^129^^,^^130^^,^^131^ In recent years, SDT has shown great potential for the treatment of brain tumors.^16^ Liu et al. synthesized a multifunctional nanozyme that can target manganese dioxide to glioma cells and even mitochondria, thereby catalyzing high levels of hydrogen protons (H), H^+^_2_O_2_, and glutathione (GSH) in the microenvironment. It continuously produces oxygen and consumes GSH to change the redox level and generate ROS under LIFU illumination.^132^ In an *in vitro* malignant glioma cell model, 5-aminolevulinic acid (5-ALA) was converted to protoporphyrin IX (PPIX) by FUS combined with the SDT of 5-ALA to increase the level of ROS and cause cell death.^133^ Animal studies have also shown that FUS combined with the systemic administration of 5-ALA can effectively treat intracranial glioma and prolong survival in rats.^134^ In addition to 5-ALA, some *in vitro* studies have used sinoporphyrin sodium purified from photofrin II as a sound sensitizer. This sound sensitizer can enter cancer cells, accumulate in mitochondria, produce ROS to increase cytotoxicity, and harm human glioblastoma cells, resulting in potent antitumor effects.^135^

## Sonobiopsy

For many years, brain tumors were generally confirmed by the discovery of possible tumor lesions through imaging examinations (such as MRI and computed tomography) and surgical resection for tissue biopsy. However, invasive brain tumor resection is associated with surgical risk^136^ and can cause complications such as bleeding, infection, and nerve injury.^137^^,^^138^ In addition, some patients cannot tolerate invasive procedures.^139^ Therefore, noninvasive methods for detecting brain tumors are urgently needed. Blood-based liquid biopsy is increasingly used as a noninvasive diagnostic method, but, due to its low detection rate, its clinical application in brain tumors is limited. FUS-enabled liquid biopsy (sonobiopsy) is an emerging technique that uses FUS to promote the release of tumor markers into the circulatory system and cerebrospinal fluid (CSF), thus facilitating tumor detection. FUS can temporarily open the BBB, enhancing the transport of molecules in and out of the microenvironment and enhancing communication between the brain TME and blood.^140^^,^^141^

**Figure 4:**
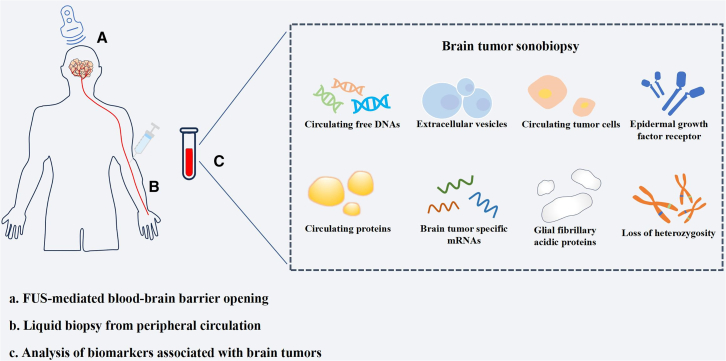
Brain tumor sonobiopsy Focused ultrasound-enabled liquid biopsy (sonobiopsy) uses focused ultrasound to promote the release of tumor biomarkers into the circulatory system, thus facilitating tumor detection.

A tumor excretes its contents into the blood^142^ and CSF.^143^ These contents include circulating tumor DNA, tumor-specific mRNAs, microRNAs, circulating tumor cells, extracellular vesicles, proteins,^144^ and cell-free DNA (cfDNA),^145^ among other substances, which can provide information for noninvasive biopsy. Blood-based liquid biopsy can rapidly reveal clinically relevant tumor information.^146^ However, the existence of the BBB prevents the release of brain tumor biomarkers into the peripheral circulation.^147^ FUS combined with microbubbles can destroy tight junctions through cavitation, increase the permeability of the BBB, and reversibly open the BBB,^148^ thereby increasing the release of brain tumor biomarkers. In an animal study in which glioblastoma cells were transfected with enhanced green fluorescent protein (eGFP), eGFP mRNA in the circulating blood was significantly increased after FUS treatment.^137^ Furthermore, in a human trial of 9 patients, plasma cfDNA, neuron-derived extracellular vesicles, and S100-calcin B were increased 2.6-, 3.2-, and 1.4-fold, respectively, after MRgFUS intervention approximately 30 min after the last sonication, and no patients experienced serious adverse events.^147^ Therefore, marker biopsy based on FUS-mediated BBB opening actively enhances information exchange between the brain TME and the circulatory system and has great application prospects in the noninvasive diagnosis of brain tumors (Figure 4). A comprehensive elucidation of sonobiopsy’s technical framework and its translational implications in characterizing the brain TME has been systematically addressed in our prior publication.^141^

## Clinical application

Currently, numerous clinical trials for the diagnosis and treatment of brain tumors using FUS are underway. These studies are mainly focusing on the effects of FUS on the microenvironment, involving human BBB opacity, liquid biopsy, and SDT (Table 2).

**Table 2:** Clinical trials for the treatment of brain tumors via FUS

NCT number	Purpose of research	Status	Conditions	Interventions
NCT04940507	safety and feasibility of liquid biopsy	recruiting	brain neoplasms	1. partial ablation of brain tumor using Exablate Neuro 4000 device (InSightec Ltd, Tirat Carmel, Israel) and blood and CSF draws for liquid biopsy 2. ablation of ventral intermediate nucleus of the thalamus (VIM) with MRgFUS using Exablate Neuro 4000 device (InSightec Ltd, Tirat Carmel, Israel) and blood draws
			liquid biopsy	
NCT05733312	extracellular impact of blood-brain barrier disruption (BBBD)	recruiting	brain tumors	device: InSightec’s Exablate Neuro Model 4000 Type 2.0 (220 kHz) system
NCT05755399	brain tumor imaging	recruiting	cranial neurosurgery	device: brain imaging using transcranial focused ultrasound (tFUS)
NCT05630209	safety and efficacy of BBBD for doxorubicin delivery	recruiting	brain tumors	device: Exablate Model 4000 Type 2.0/2.1
NCT05615623				drug: doxorubicin
NCT05317858	BBBD combined with standard-of-care treatment of NSCLC brain metastasis	recruiting	glioblastoma	device: Exablate Model 4000 Type 2.0/2.1 drug: pembrolizumab
			glioma	
			liquid biopsy	
NCT05383872	BBBD for liquid biopsy	recruiting	brain tumors	device: Exablate Model 4000
NCT03028246	feasibility and safety of ablation	recruiting	benign centrally located intracranial tumors	device: Exablate 4000 system
NCT05762419	BBBO for drug delivery	recruiting	diffuse intrinsic pontine glioma	drug: etoposide; oral, 50 mg
			diffuse midline glioma, H3 K27M-mutant	device: focused ultrasound with neuro-navigator-controlled sonication
NCT06039709	safety and feasibility of sonodynamic therapy	recruiting	recurrent glioblastoma	5-ALA and LIFU
			glioblastoma multiforme	
			GBM	
NCT04804709	BBBO for drug delivery	active, not recruiting	diffuse intrinsic pontine glioma	Drug: panobinostat 15 mg device: focused ultrasound with neuro-navigator-controlled sonication
			diffuse pontine and thalamic gliomas	
			diffuse midline glioma, H3 K27M-mutant	
NCT05123534	safety and tolerability of sonodynamic therapy	recruiting	diffuse intrinsic pontine glioma	SONALA-001 (ALA) and Exablate 4000 Type 2.0 MR-guided focused ultrasound
			diffuse midline glioma	

## Strengths and limitations

There are other brain tumor treatment techniques that have been used in research and clinical practice, such as laser interstitial thermal therapy (LITT),^149^^,^^150^^,^^151^ photodynamic therapy (PDT),^152^^,^^153^^,^^154^^,^^155^ intraoperative brachytherapy (IOBT),^156^^,^^157^^,^^158^ and tumor-treating fields (TT Fields).^159^^,^^160^^,^^161^ Comparative analysis of neuro-oncological interventions (Table 3) highlights distinctive advantages of FUS over conventional modalities. Unlike LITT and PDT requiring invasive instrumentation, FUS achieves therapeutic effects noninvasively through multi-modal mechanisms—combining thermal ablation for tumor debulking with immunologically active cavitation effects.^162^ Its unique BBB/BTB modulation capacity enables simultaneous diagnostic sonobiopsy and enhanced drug delivery, features absent in IOBT’s localized radiation or TT Fields’ mitosis-focused approach.^163^ Crucially, FUS-mediated TME remodeling (permeability enhancement, interstitial pressure reduction, and immune activation) demonstrates synergistic potential with adjuvant therapies—a paradigm distinct from the monotherapeutic limitations of laser-based or radiation techniques. These attributes position FUS as a versatile platform technology for precision neuro-oncology.

**Table 3:** Comparative analysis of FUS with other neuro-oncological therapeutic modalities

Parameter	FUS (focused ultrasound)^162^^,^^163^	LITT (laser interstitial thermal therapy)^149^^,^^150^^,^^151^	PDT (photodynamic therapy)^152^^,^^153^^,^^154^^,^^155^	IOBT (intraoperative brachytherapy)^156^^,^^157^^,^^158^	TT fields (tumor-treating fields)^159^^,^^160^^,^^161^
Application	noninvasive tumor ablation, BBB opening, sonobiopsy	localized tumor ablation via thermal coagulation	tumor cell death via photosensitizer activation	radiation therapy during surgery	noninvasive antitumor electric field application
Mechanism	thermal ablation (hyperthermia) or mechanical cavitation	hyperthermia-mediated protein denaturation	oxidative damage from light-activated ROS	localized radioactive isotope irradiation	alternating electric fields disrupting mitosis
Medical equipment	phased-array transducer, coupling system	laser-emitting optical fiber	optical fiber + photosensitizer	radioactive seeds (Cs-131, I-125, Ir-192)	insulated transducer arrays
Spatial resolution	0.5–3 mm (skull-dependent, requires correction)	2–5 mm (requires precise fiber placement)	∼1 cm (limited by light penetration)	<1 mm (precise localized radiation)	low (dependent on field distribution)
Invasive/noninvasive	noninvasive	minimally invasive (catheter insertion)	minimally invasive	invasive (surgical implantation)	noninvasive
Tumor response	cell death, immunomodulation	coagulative necrosis, apoptosis	apoptosis	radiation necrosis	mitotic arrest, autophagy, impaired DNA repair
TME effects	enhanced BBB/BTB permeability, immunostimulation	microvascular damage, oxidative stress	immune activation, hypoxia reduction	vascular damage, radiosensitization	reduced angiogenesis, immunomodulation
Key limitations	skull attenuation, requires temperature monitoring	risk of peritumoural thermal injury, limited deep-tumor access	oxygen dependency, poor tissue penetration	long-term radiation exposure, surgical risks	limited efficacy in bulky tumors, patient compliance

Despite its potential in basic research and clinical application, FUS also has several limitations. In terms of its thermal effect, achieving both safe ablation and complete ablation is sometimes difficult. Therefore, focal ablation may lead to incomplete ablation and subsequent recurrence.^164^ With respect to mechanical effects, since complex parameter combinations are involved in ultrasonic therapy (frequency, intensity, time, etc.), controlling the cavitation effect in the tissue is challenging.^165^ Moreover, brain tumors are very heterogeneous. For example, gliomas can be divided into 13 types according to different classification criteria^166^^,^^167^ and dozens of subgroups according to proteome variability analysis.^29^ Different subtypes have different microenvironments. This is a challenge for FUS treatment of brain tumors, which should fully consider the heterogeneity of brain tumors and the microenvironmental characteristics of different subtypes.

## Conclusions

In summary, FUS represents a promising diagnostic and therapeutic modality for brain tumors, demonstrating clinical value through its multifactorial modulation of the TME. Mechanistically, FUS alters tumor survival conditions via coordinated effects on immune-inflammatory responses, oxidative stress mitigation, BBB/BTB permeability enhancement, targeted drug delivery optimization, and image-guided biomarker sampling. While animal studies have robustly validated these preclinical benefits, notable limitations persist: current FUS protocols face challenges in achieving consistent transcranial focal precision, and potential thermal bioeffects require stricter control paradigms for clinical adaptation. Phase 1/2 clinical trials remain urgently needed to establish safety profiles and efficacy benchmarks across heterogeneous patient populations. Alternative modalities like LITT and convection-enhanced delivery may synergize with FUS for deeper-seated lesions where acoustic accessibility proves suboptimal. As a noninvasive platform, FUS holds transformative potential for neuro-oncology management, contingent upon resolution of technological constraints through interdisciplinary engineering refinements.

## Acknowledgments

This work was supported by the 10.13039/501100001809National Natural Science Foundation of China (32071316 and 32211530049), the Key Research and Development Project of Shaanxi Province (2023-YBSF-121), and the Practice and Innovation Funds for Graduate Students of Northwestern Polytechnical University (PF2024113).

## Author contributions

L.Y. conceived and designed the study. K.F. and H.H. wrote the paper. L.Y. provided supervision. X.Z. and L.L. reviewed and revised the manuscript. All the authors contributed to the article and read and approved the submitted version.

## Declaration of interests

The authors declare no competing interests.
